# Advances on liver cell‐derived exosomes in liver diseases

**DOI:** 10.1111/jcmm.16123

**Published:** 2020-11-27

**Authors:** Yan Jiao, Ping Xu, Honglin Shi, Dexi Chen, Hongbo Shi

**Affiliations:** ^1^ Beijing Institute of Hepatology Beijing Youan Hospital Capital Medical University Beijing China; ^2^ Beijing Engineering Research Center for Precision Medicine and Transformation of Hepatitis and Liver Cancer Beijing China

**Keywords:** biomarker, exosome, hepatocyte, liver disease

## Abstract

Exosomes are extracellular vesicles with diameters ranging from 30 to 150 nm, which contain several donor cell‐associated proteins as well as mRNA, miRNA, and lipids and coordinate multiple physiological and pathological functions through horizontal communication between cells. Almost all types of liver cells, such as hepatocytes and Kupffer cells, are exosome‐releasing and/or exosome‐targeted cells. Exosomes secreted by liver cells play an important role in regulating general physiological functions and also participate in the onset and development of liver diseases, including liver cancer, liver injury, liver fibrosis and viral hepatitis. Liver cell‐derived exosomes carry liver cell‐specific proteins and miRNAs, which can be used as diagnostic biomarkers and treatment targets of liver disease. This review discusses the functions of exosomes derived from different liver cells and provides novel insights based on the latest developments regarding the roles of exosomes in the diagnosis and treatment of liver diseases.

## 1 INTRODUCTION

Exosomes are primarily derived from multi‐vesicular bodies, which fuse with the plasma membrane and subsequently release internal vesicles in the form of exosomes.^1^ Exosomes are naturally closed vesicles with lipid bilayers. Electron microscopy has shown them to have disc‐ or cup‐shaped structures with, diameters ranging from 30 to 150 nm. Almost all types of cells in the human body can release exosomes, including reticulocytes, tumour cells and mesenchymal stem cells.^2^ As a substance carrier, exosomes contain a variety of biologically active molecules, including lipids, proteins and nucleic acids, such as mRNA, microRNA (miRNA) and long non‐coding RNA (lncRNA). Established exosome markers include CD63, syntenin‐1, TSG101 and integrin among others. Recent studies have shown that exosomes can serve as potential tools for diagnosis and treatment owing to their ability to carry functional RNA or small molecules. In addition, the contents of exosomes can be selectively manipulated using various methods, which can further help develop treatment strategies.

## 2 PHYSIOLOGICAL FUNCTIONS OF EXOSOMES

Among the extensive physiological functions of exosomes, the most important is its role in information exchange and intercellular material transfer.^3^ Exosomes communicate with cells using three major mechanisms: binding to receptors on target cells, fusing directly with target cell membranes and entering target cells by endocytosis. Endocytosis can occur by clathrin‐dependent and clathrin‐independent mechanisms.^4^, ^5^ Exosomes contain several types of bioactive molecules. Lipids are essential for maintaining the morphological stability of exosomes in extracellular fluids, protecting exosomes from enzymatic degradation^6^ and participating in multiple biological processes as signal molecules. Proteins present in exosomes can be divided into two categories. Non‐specific proteins, such as cytoskeletal proteins, four‐transmembrane proteins (CD9, CD63) and heat‐shock proteins (such as HSP90), are present in all exosomes,^7^ whereas specific proteins are those associated with the source cells of exosomes specifically. For example, exosomes derived from tumour cells carry large quantities of tumour antigens, which may be related to cellular signal transduction.^8^, ^9^ In addition, exosomes contain different nucleic acids, such as mRNA, miRNA and lncRNA, which are considered potential markers for the diagnosis of disease.^10^

## 3 EXOSOMES DERIVED FROM LIVER CELLS

**Figure 1:**
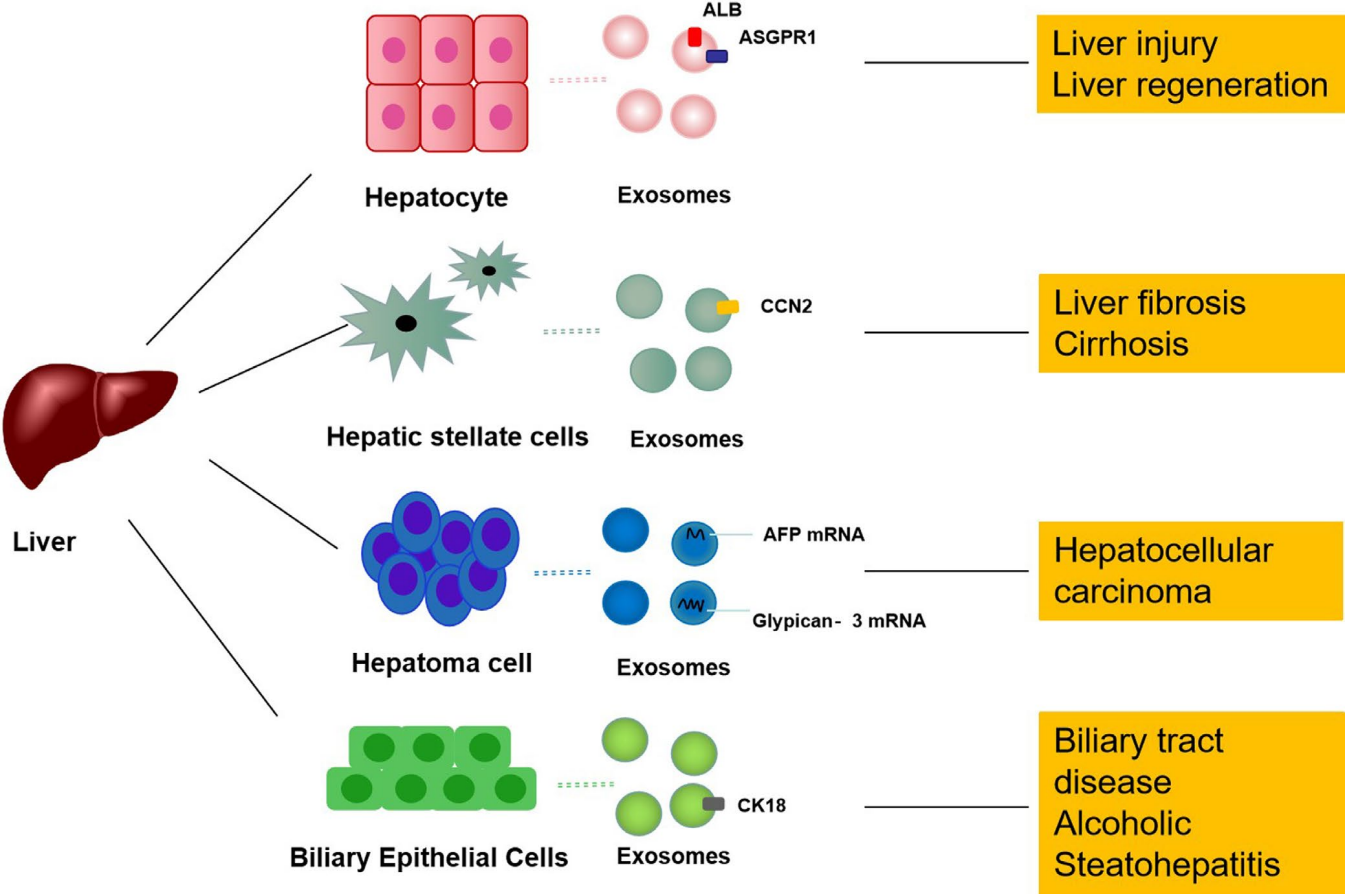
Exosomes derived from liver cells are involved in the pathogenesis of liver diseases and may serve as diagnostic markers and therapeutic targets. Liver cell‐derived exosomes carry liver‐specific proteins and miRNAs, which can be used as potential biomarkers and targets in liver disease. For example, albumin is encapsulated by exosomes secreted by hepatocytes and contributes to liver injury as well as to liver regeneration

The liver contains hepatocytes, hepatic stellate cells (HSCs) and Kupffer cells, which are exosome‐releasing/‐targeted cells. Exosomes contain tissue‐specific proteins and miRNAs derived from source cells, and the number and content of exosomes may fluctuate based on the specific disease state. Liver cell‐derived exosomes carry liver‐specific proteins and miRNAs, such as carboxylesterase‐1 (CES1), alcohol dehydrogenase‐1 (ADH1), glutathione S‐transferase, apolipoprotein A‐1 (APOA1), albumin (ALB), haptoglobin (HP) and miRNA‐122,^11^ which can be used as potential biomarkers and targets in liver disease. ALB and ASGPR1 are encapsulated in exosomes secreted by hepatocytes ^12^, ^13^ and participate in liver injury as well as liver regeneration. Exosomes derived from liver cancer cells containing alpha fetoprotein (AFP) mRNA and glypican‐3 mRNA are used for the diagnosis and treatment of liver cancer.^14^ Exosomes derived from HSCs carrying connective tissue growth factor (CCN2) participate in the induction of liver fibrosis.^15^ Cytokeratin 18 (CK18) is present in exosomes derived from bile duct cells and is used to diagnose biliary diseases, alcoholic hepatitis (AH) and cirrhosis ^16^, ^17^ (Figure 1).

### 3.1 Hepatocyte‐derived exosomes

**Figure 2:**
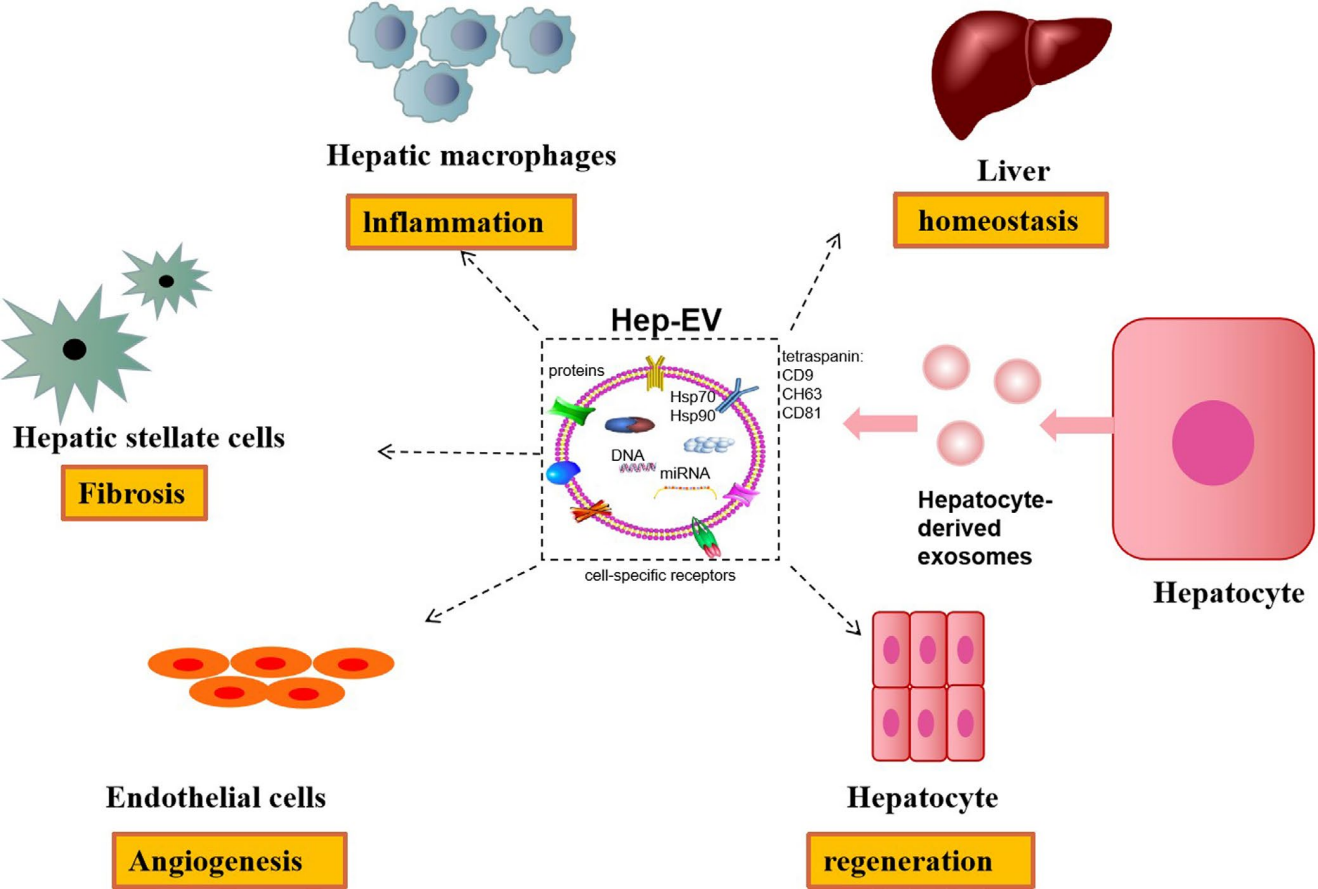
Functions of exosomes derived from hepatocytes. The figure shows the bioactivities of hepatocyte‐derived exosomes, including homoeostasis, angiogenesis, tissue repair and regeneration, inflammation, and fibrosis

Studies have shown that hepatocyte‐derived exosomes carrying hepatocyte‐specific contents can easily pass through the sinusoidal endothelium.^18^, ^19^ They stimulate various non‐parenchymal cells (Figure 2), including monocytes,^20^ lymphocytes,^21^ HSCs^22^, ^23^ and endothelial cells,^24^ and play an important role in signalling transmission.

To date, a large number of studies have shown that hepatocyte‐derived exosomes are involved in the pathogenesis of liver diseases. Momen‐Heravi et al showed that, in a model of AH, miRNA‐122 was present in exosomes released by alcohol‐treated hepatocytes. Exosomes were absorbed by the human monocytic cell line THP‐1 and were observed to transfer miRNA‐122 into THP‐1 cells. miR‐122 enhances the inflammatory response in monocytes and affects their immune function.^20^ In alcoholic liver disease, alcohol‐exposed human monocytes can release exosomes, which can subsequently stimulate the polarization of naive monocytes, which then form M2 macrophages.^25^ Alcohol stimulates the expression of miR‐155 in hepatocytes. This miR‐155 targets mammalian target of rapamycin (mTOR), Ras homolog enriched in brain (Rheb), lysosomal‐associated membrane protein 1 (LAMP1) and lysosomal‐associated membrane protein 2 (LAMP2); it also disrupts autophagy at the lysosomal level and increases exosome release.^26^ In a hepatitis C virus (HCV)‐induced fibrosis model, exosomes released by HCV‐infected hepatocytes carry miR‐19a, which can be internalized by HSCs. miR‐19 was observed to target suppressor of cytokine signalling 3 (SOCS3) in HSCs and activate the STAT3/TGFβ‐1/Smad3 signalling pathway to convert resting HSCs to activated HSCs.^27^

Exosomes play an important role in liver injury and regeneration. Holman et al used hepatocyte‐derived exosomes to treat THP‐1 cells for 24 hours and followed this by stimulation of the cells with lipopolysaccharide (LPS) for 6 hours; they observed that the levels of interleukin (IL)‐1β and IL‐8 produced in response to stimulation by LPS reduced and that genes encoding proteins associated with the innate immune response were significantly down‐regulated. Hepatocyte‐derived exosomes could transfer functional miRNAs and multiple immune‐mediated transcripts to monocytes to inhibit the release of cytokine stimulated by LPS.^28^, ^29^ In addition, hepatocyte‐derived exosomes were observed to promote the proliferation of hepatocytes in vitro and liver regeneration in vivo by mediating the transfer of sphingosine kinase 2 (SK2) to target cells and inducing up‐regulation of intracellular sphingosine‐1‐phosphate (S1P).^30^, ^31^

### 3.2 HSC‐derived exosomes

Under normal conditions, HSCs remain in the resting state, and their activation is closely related to liver fibrosis. Studies have shown that HSC‐derived exosomes promote fibrosis. When HSCs are activated, they can release exosomes containing CCN2, which is transmitted between HSCs. Exosomal CCN2, along with other fibrosis‐related molecules, may amplify fibrogenic signalling to promote hepatic fibrosis.^15^ Wan et al showed that exosomes derived from activated HSCs contain glycolysis‐related molecules, such as glucose transporter 1 (GLUT1) and pyruvate kinase M2 (PKM2). These exosomes are internalized by resting HSCs, macrophages and liver sinusoidal endothelial cells. The increase in the intracellular levels of GLUT1 and PKM2 induced non‐parenchymal cell activation and metabolic conversion in the liver.^32^ Other experiments also supported the observation that exosomes produced by HSCs promote fibrosis by acting on HSCs or other non‐parenchymal cells. Platelet‐derived growth factor (PDGF)‐BB is a key molecule in the process leading to liver fibrosis. Kostallari et al reported that PDGF‐BB–treated HSCs release PDGF receptor‐alpha (PDGFRα)‐enriched exosomes using an Src homology 2 domain tyrosine phosphatase 2‐dependent mechanism. These exosomes promote HSC migration and liver fibrosis. Interference with PDGFRα in exosomes was shown to suppress carbon tetrachloride (CCl_4_)‐induced liver fibrosis in mice.^33^

In addition, exosomes derived from HSCs contribute to the pathology of liver cancer. Researchers showed that exosomes derived from HSCs deliver miR‐335‐5p to recipient hepatocellular carcinoma (HCC) cells, inhibiting the proliferation and in vitro invasion of HCC cells and inducing the shrinkage of HCC tumours in vivo.^34^ Moreover, researchers observed that the expression of miR‐30a was down‐regulated in exosomes derived from activated HSCs, which may prevent HSC activation by suppressing autophagy.^35^

### 3.3 Cholangiocyte‐derived exosomes

Cholangiocyte‐derived exosomes are closely associated with the onset of cholestatic liver injury, and exosomes containing lncRNA‐H19 are key contributors. Researchers found that, in multidrug resistance‐associated protein 2 knockout mice, exosomes secreted by cholangiocytes can transfer lncRNA‐H19 to hepatocytes; this subsequently inhibits the expression of hepatic small heterodimer partner, interferes with bile acid homoeostasis and promotes cholestatic liver injury.^36^ Research showed that exosomes containing lncRNA‐H19 are also major contributors to liver fibrosis. HSCs are the primary target cells of cholangiocyte‐derived exosomes. Particularly in cholestasis, HSCs preferentially take up exosomes secreted by cholangiocytes. lncRNA‐H19 promotes the proliferation and activation of HSCs by increasing G1/S cell cycle transition and up‐regulates fibrotic gene expression in fibroblasts derived from HSCs to promote liver fibrosis.^37^

Certain studies have also shown that exosomes accumulate in the lumen of the intrahepatic bile duct and bile exosomes interact with cholangiocyte cilia; these phenomena affect intracellular and extracellular regulated protein kinase signalling, up‐regulate miR‐15A expression and inhibit cholangiocyte proliferation. Thus, the interaction of bile exosomes with cholangiocyte cilia could be a novel mechanism underlying intercellular communication in the liver.^38^

### 3.4 HCC cell‐derived exosomes

Liver cancer‐derived exosomes are involved in several pathological processes (Table 1). Exosomes can be transferred horizontally between different liver cancer cells and change the original biological functions of the cells. For example, Huh7 cell‐derived exosomes can transfer miR‐122 to HepG2 cells to inhibit the growth and accelerate the ageing process in HepG2 cells.^39^ HCC cell‐derived exosomes also affect tumour angiogenesis. miR‐210‐containing exosomes released by HCC cells can be transferred to endothelial cells, preventing angiogenesis by inhibiting the expression of mothers against decapentaplegic homolog 4 (SMAD4) and signal transducer and activator of transcription 6 (STAT6).^40^ HCC cell‐derived exosomes also participate in metastasis in HCC. Hepatoma cell‐derived exosomal miRNA‐103 was observed to target junction proteins, which attenuated the integrity of the endothelial junction and promoted tumour metastasis.^41^ Fang et al^42^ reported that highly metastatic HCC cells secrete exosomal miR‐1247‐3p, which activates β1‐integrin‐NF‐κB signalling in fibroblasts, and cancer‐associated fibroblasts promote lung metastasis of liver cancer by secreting pro‐inflammatory cytokines. In addition, exosomes also contribute to liver cancer progression. Cheng et al^43^ reported that p120‐catenin (p120ctn) present in exosomes secreted by liver cancer cells inhibited the growth of liver cancer stem cells and the proliferation and metastasis of HCC cells through the STAT3 pathway.

**Table 1:** Components of exosomes derived from hepatocellular carcinoma cells and their roles

Component	Role	Reference	Year of publication	Reference no.
miR‐1247‐3p	Induces cancer‐associated fibroblast activation	Fang et al	2018	42
miR‐18a	Increased level/diagnostic marker	Sohn et al	2015	44
miR‐103	Metastasis	Fang et al	2018	41
miR‐221	Increased level/diagnostic marker	Sohn et al	2015	44
miR‐222	Increased level/diagnostic marker	Sohn et al	2015	44
miR‐224	Increased level/diagnostic marker	Sohn et al	2015	44
miR‐210	Promotes angiogenesis	Lin et al	2018	40
miR‐122	Improve treatment effects	Lou et al	2015	52
miR‐638	Predict the prognosis of HCC	Shi et al	2018	46
miR‐335	Novel therapeutic agent	Wang et al	2018	34
circPTGR1	Increased level/ prognosis marker	Wang et al	2019	49
LINC00635	Diagnosis and Prognosis of HCC	Xu et al	2018	48
LINC00161	Increased level/ prognosis marker	Sun et al	2018	50

Abbreviation: HCC, hepatocellular carcinoma.

## 4 EXOSOMES AS DIAGNOSTIC AND TREATMENT TOOLS FOR LIVER DISEASE

**Figure 3:**
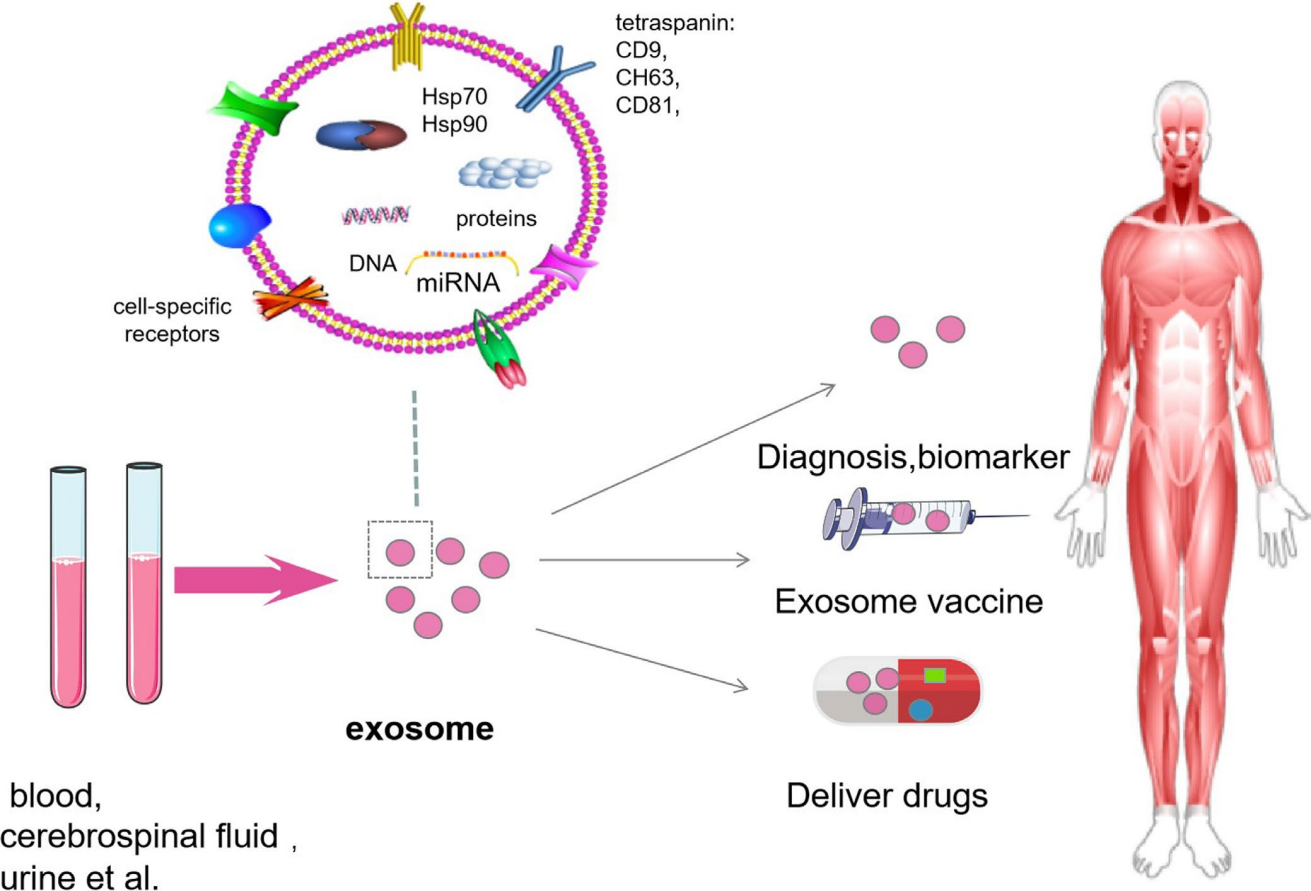
Exosomes as novel tools for the treatment and diagnosis of diseases. Exosomes are present in a variety of body fluids, such as blood, urine and cerebrospinal fluid, and can be used as potential diagnostic biomarkers. Genetically engineered exosomes may serve as novel vaccines. In addition, exosomes are important non‐toxic carriers and can be used as drug delivery tools

Findings from certain studies suggest that there are differences in the number and content of exosomes released at different stages of liver disease. Exosomes are advantageous for diagnosis, as they offer the specificity of liver biopsy samples and the non‐invasiveness of peripheral blood samples, which are potential biomarkers of liver disease (Table 2). Recently, researchers have selectively manipulated the contents of exosomes to develop novel strategies for disease treatment. Exosomes are important non‐toxic carriers and elicit low immunogenicity and toxicity. They can be used as drug delivery tools in the treatment of certain liver diseases (Table 3) (Figure 3).

**Table 2:** Exosomes are potential biomarkers of liver disease

Disease	Maker	Reference no.
Primary hepatic carcinoma	miR‐18a, miR‐221, miR‐222, miR‐224, miR‐101, miR‐106b, miR‐122, miR‐195, miR‐30d, miR‐140, miR‐29b, miR‐638, lncRNAXist, circPTGR1 LNCRNAs ENSG00000258332.1 and LINC00635	44, 45, 46, 47, 48, 50
NAFLD and ALD	miR‐192‐5P, ceramide and S1P, CD40L, miRNA‐192 and miRNA‐30a	59, 60, 61, 62
Acute liver injury and liver failure	miRNA‐122, miRNA‐155, miRNA‐192, albumin, fibrinogen B, Gnb2l, haptoglobin, Rbp4	11, 70, 71
Liver fibrosis	CCN2, GLUT1, PKM2, PDGFRα, miR‐30a	15, 32, 33, 35

Abbreviations: ALD, alcoholic liver disease; CCN2, connective tissue growth factor; GLUT1, glucose transporter 1; Gnb2l, G protein β‐polypeptide 2‐like 1; NAFLD, non‐alcoholic fatty liver disease; PDGFRα, PDGF receptor‐alpha;PKM2, pyruvate kinase M2; Rbp4, retinol‐binding protein 4; S1P, sphingosine‐1‐phosphate.

**Table 3:** Roles of exosomes in the treatment of liver disease

Disease	Donor cells	Recipient cells	Mediators	Implications	Reference no.
Primary hepatic carcinoma	Macrophage	HCC	miR‐142 miR‐223	Inhibit tumour cell proliferation and growth	51
	AMSC	HCC	miR‐122	Increase sensitivity to chemotherapy drugs	52
	293T cell	Liver cancer cell	miR‐26a	Inhibits the cell cycle and cell proliferation	53
	HCC	Dendritic cell	HCC antigens	Trigger DC‐mediated immune response	54
	HSC	HCC	miR‐335‐5p	Induce the shrinkage of HCC tumours	34
Liver fibrosis	AMSC	HSC	miR‐122	Ameliorate liver fibrosis	52
	Amnion‐MSC	KC/HSC	Not mentioned	Reduce HSC and KC activation/ liver fibrosis	55
	HUC‐MSC	Mice with liver fibrosis	Not mentioned	Ameliorate CCl4‐induced liver fibrosis	56
	BM‐MSC	Mice with liver fibrosis	Not mentioned	Inhibit HSC activation	57
	Hepatocyte	HSC	miRNA‐192	Induce HSC transdifferentiation into myofibroblasts	58
	Hepatocyte	HSC	Not mentioned	Activate TLR3 in HSCs and exacerbate fibrosis	22
NAFLD and ALD	Hepatocyte	Macrophage	Apoptosis‐inducing ligand	Activate inflammatory phenotype in macrophage	64
	Hepatocyte	Macrophage	CXCL10	Induce macrophage chemotaxis	65
	Obese mice	Lean mice	miRNA27a‐3pmiRNA‐192 miRNA‐122	Modulate glucose and lipid metabolism in mice	66
	Hepatocyte	KC	mtdsRNA	Trigger the production of IL‐1β from KCs	68
Acute liver injury and liver failure	BM‐MSC	Murine model of hepatic IRI	Not mentioned	Reduce liver damage by regulating inflammatory responses	72
	BM‐MSC	Hepatocyte	Not mentioned	Improve survival in hepatic failure mice	73
	MSC	Hepatocyte	Not mentioned	Promote hepatic regeneration	74
	AMSC	Macrophage	miR‐17	Alleviate LPS/GalN induced liver failure	75
	CP‐MSC	HSC	miR‐125b	Ameliorate liver fibrosis	76
Viral hepatitis	Macrophage	Hepatocyte	IFN‐α‐induced antiviral substances	Exosomes exploit virus entry machinery for access to hepatocyte	84
	Monocyte	Healthy mice	Not mentioned	Promote inflammatory response	86
	Not mentioned	Hepatoma cell/ Hepatocyte	Viral envelope glycoprotein E2	Make HCV less sensitive to antibody neutralization	77
	Not mentioned	Antigen‐presenting cell	Nef^mut^‐based DNA vectors exosomes	Induce CTL immunization against full‐length antigens	87

Abbreviations: ALD, alcoholic liver disease; AMSC, adipose‐derived mesenchymal stem cell; AMSC, amnion‐derived MSC; AMSCs, adipose tissue‐derived mesenchymal stem cells. CP‐MSC, chorionic plate‐derived mesenchymal stem cell; BM‐MSC, bone marrow mesenchymal stem cell; CTL, cytotoxic T lymphocyte; CXCL10, CXC motif chemokine ligand‐10; HCCs, hepato‐carcinoma cells; HCV, hepatitis C virus; HUC‐MSC, human umbilical cord mesenchymal stem cell; KC, Kupffer cells; LNPCs, liver non‐parenchymal cells; MSC, mesenchymal stem cell; mtdsRNA, mitochondrial double‐stranded RNA; NAFLD, non‐alcoholic fatty liver disease.

### 4.1 Primary hepatic carcinoma

#### 4.1.1 Biomarkers

Several studies have revealed that certain differentially expressed exosomal miRNAs can be used as potential biomarkers for diagnosing HCC. Sohn et al showed that the serum levels of exosomal miR‐18a, miR‐221, miR‐222, and miR‐224 in patients with HCC are significantly higher than those in patients with chronic hepatitis B (CHB) infection, while the serum levels of miR‐101, miR‐106b, miR‐122 and miR‐195 are lower than those in CHB patients. These exosomal miRNAs may serve as novel serum markers for the diagnosis of HCC.^44^ Exosomal miRNA can also be used as prognostic markers of liver cancer. miR‐30d, miR‐140 and miR‐29b are significantly associated with survival in patients with HCC and may be used as biomarkers for predicting the migration of HCC cells and HCC prognosis and may guide the treatment of advanced HCC.^45^ In addition, certain miRNAs can be used as indicators for predicting the risk of relapse clinical conditions. The level of exosomal miR‐638 is inversely related to tumour size, vascular infiltration and tumour node metastasis stage. The serum levels of exosomal miR‐638 were reduced in patients with HCC, and these patients exhibited lower overall survival than patients with higher levels of exosomal miR‐638.^46^ In addition to miRNA, non‐coding exosomal RNAs can also be used as potential markers. Ma et al^47^ demonstrated that exosomes mediate the regulation of lncRNA X inactivation‐specific transcript (Xist) expression in blood cells, which indicates that Xist expressed by monocytes and granulocytes may serve as a valuable biomarker for the diagnosis of liver cancer in women. Xu et al^48^ reported that the measurement of the levels of serum exosomal lncRNA ENSG00000258332.1, serum exosomal RNA LINC00635 and serum AFP might be a promising method for the diagnosis and prognosis of HCC. Other studies have revealed that, compared to normal patients, patients with HCC exhibit significant up‐regulation of LINC00161 and circPTGR1 (a type of circRNA), which indicates that these can be used for clinical staging and prognosis.^49^, ^50^

#### 4.1.2 Therapy

There exists mutual communication between exosomes and their targeted tumour cells. The delivery of exosomal miRNA and lncRNA significantly inhibits cancer development and leads to antitumour effects. For example, miR‐142 and miR‐223 can be transferred from human macrophages to hepatocytes via exosomes to inhibit tumour cell proliferation and growth.^51^ Lou et al showed that miR‐122 can negatively regulate the expression of target genes, such as cyclin B1 and insulin‐like growth factor 1 receptor genes. Adipose tissue‐derived mesenchymal stem cells transfected with miR‐122 can transport miR‐122 to HCC cells via exosomes to promote apoptosis and cell cycle arrest and reduce liver fibrosis, as well as to increase the sensitivity of HCC cells to chemotherapy drugs.^52^ Engineered exosomes have been studied extensively in recent years. Liang et al^53^ reported that modified 293T cells were observed to release ApoA1‐CD63‐expressing exosomes loaded with miR‐26a by electroporation and that the exosomes were internalized by HepG2 cells via scavenger receptor class B type 1 receptor‐mediated endocytosis, which up‐regulated the expression of miR‐26a, a key molecule that inhibits the cell cycle and cell proliferation. In addition, exosomes can be used as cancer vaccines. Dendritic cells (DCs) bind HCC tumour cell‐derived exosomes by increasing the number of activated T cells and interferon (IFN)‐γ levels as well as by reducing the expression of the anti‐inflammatory cytokines IL‐10 and TGF‐β to induce cellular immune response. This process promotes tumour suppression and can be used as a new strategy for the development of cancer vaccines.^23^, ^54^

### 4.2 Liver fibrosis

#### 4.2.1 Biomarkers

Hepatic fibrosis occurs owing to the suppression of liver cell regeneration. It is a process for scar repair induced by the accumulation and precipitation of components, such as type I collagen fibres. Exosomal elements play an important role in liver fibrosis (Table 2).

#### 4.2.2 Therapy

At present, most treatments for liver fibrosis that involve the use of exosomes require hepatocytes and progenitor cells. There are several potential treatment modalities based on this. Researchers have demonstrated that miRNA‐122 loaded in exosomes derived from mesenchymal stem cells (MSCs) can inhibit the activation and proliferation of primary HSCs, and continued treatment for 4 weeks using this method can improve CCl_4_‐induced liver fibrosis in mice.^52^ Ohara et al^55^ demonstrated that amniotic mesenchymal stem cell‐derived extracellular vesicles in primary cell culture can suppress the activation of Kupffer cells and HSCs and can improve CCl_4_‐induced liver fibrosis. Exosomes derived from human umbilical cord mesenchymal stem cells (HUC‐MSCs) also reduce fibrosis by inhibiting the expression of collagen and TGF‐β1 in vivo.^56^ In addition, MSC‐derived exosomes suppress liver fibrosis by improving liver function and inhibiting inflammation and HSC activation.^57^ In addition to the use of stem cells and progenitor cells, in a model of CCl_4_‐induced liver injury, hepatocyte‐derived exosomes were observed to activate Toll‐like receptor 3 (TLR3) in HSCs and promote fibrosis by enhancing IL‐17A secretion from T cells, suggesting that TLR3 may serve as a novel therapeutic target for liver fibrosis.^22^ In HCV‐induced fibrosis, HCV‐replicating hepatocytes transfer miRNA‐192 to HSCs through exosomes, activating HSCs and inducing their transdifferentiation into myofibroblasts. This suggests that exosomal miR‐192 is a major regulator and potential therapeutic target in liver fibrosis induced by HCV.^58^

### 4.3 Non‐alcoholic fatty liver disease (NAFLD) and alcoholic liver disease

#### 4.3.1 Biomarkers

Liu et al reported that, during NAFLD progression, the number of exosomes containing miR‐192‐5P in plasma was higher in patients with NAFLD and in rat models than in controls, and exosomal miR‐192‐5P derived from hepatocytes with lipotoxicity induced the activation of M1 macrophages and increased the expression of M1‐specific cytokines, such as inducible nitric oxide synthases, IL‐6 and tumour necrosis factor (TNF)‐β. This suggested that miR‐192‐5P may serve as a potential non‐invasive marker for NAFLD.^59^ In patients with non‐alcoholic steatohepatitis (NASH), the ceramide levels in peripheral blood exosomes were observed to increase significantly. The ceramide metabolite sphingosine‐1‐phosphate (S1P) activates macrophage chemotaxis. The ceramide and S1P levels in exosomes may be used as a biomarker for NASH.^60^ Alcohol exposure induces the release of CD40L‐enriched exosomes from hepatocytes, which leads to the activation of macrophages and induces inflammation in alcoholic liver disease. High levels of CD40L‐enriched exosomes have also been detected in the sera of patients with AH, which suggests that CD40L‐enriched exosomes may act as biomarkers for liver damage caused by inflammation.^61^ Researchers have shown that the number of circulating exosomes increased in alcohol‐fed mice, a model of AH. In patients with AH, the number of exosomes and the levels of miRNA‐192 and miRNA‐30a increased significantly; these are useful parameters for identifying alcohol‐induced liver damage. In particular, miRNA‐192 is a promising diagnostic marker for AH.^62^

#### 4.3.2 Therapy

Alcohol and lipotoxicity stimulate the release of exosomes from hepatocytes through different mechanisms. It has been reported, using experimental models of NASH and AH, that exosomes released by damaged or stressed hepatocytes promote disease progression by activating liver endothelial cells, HSCs and liver macrophages.^63^, ^64^, ^65^ Moreover, Castano et al^66^ demonstrated that mice fed a high‐fat diet produce circulating exosomes containing miRNAs (miRNA27a‐3p, miRNA 192, miRNA 122), which, when transfected into healthy mice, interfered with the expression of genes encoding proteins related to hepatic steatosis and inflammation. Other studies have shown that the fatty acid palmitate stimulates the release of exosomes by death receptor 5 (present on the surface of hepatocytes) that contain TNF‐related apoptosis‐inducing ligand, CXC motif chemokine ligand (CXCL)‐10, S1P and other molecules associated with macrophage activation. This is an essential step in the development of NASH. The use of Rho‐associated coiled‐coil‐containing protein kinase 1 inhibitors for suppressing the release of extracellular vesicles from hepatocytes may be a suitable treatment strategy for NASH.^67^ Lee et al reported that, in the treatment of ALD, ethanol induces the release of exosomes loaded with mitochondrial double‐stranded RNA (mtdsRNA) from hepatocytes, and the activation of TLR3 induced by mtdsRNA triggers the production of IL‐1β by the neighbouring Kupffer cells. This increases IL‐17A expression in the early stage of ALD. Therefore, TLR3 and mtdsRNA could be considered important targets for improvements in ALD treatment.^68^

### 4.4 Acute liver injury and liver failure

#### 4.4.1 Biomarkers

Acute liver injury can be categorized into physical liver injury and toxic liver injury. A different perspective for diagnosis involves monitoring the changes in exosome contents during liver injury. For example, studies have shown that miRNA‐122, miRNA‐155 and miRNA‐192 may serve as biomarkers of acute toxic liver injury.^11^, ^69^, ^70^, ^71^ In APAP‐ or galactosamine‐induced caries animal liver injury models, the number of circulating exosomes containing albumin, β‐actin, fibrinogen B, G protein β‐polypeptide 2‐like 1, haptoglobin and retinol‐binding protein 4 increased, and these were considered markers of acute liver injury.^11^, ^18^

#### 4.4.2 Therapy

In recent times, studies have shown that the injection of exosomes derived from specific types of cells can alleviate liver damage (Table 3). For example, Haga et al^72^ found that, in a mouse model of ischaemia‐reperfusion injury, exosomes derived from bone marrow mesenchymal stem cells (BM‐MSCs) reduced liver damage by regulating inflammatory responses. In a mouse model of liver failure induced by d‐galactosamine/TNF‐α, liver injury and mortality rates were reduced upon treatment with mouse and human BM‐MSC‐derived exosomes, an effect that was related to decreased inflammation.^73^ In a mouse model of liver injury induced by CCl_4_, MSC‐derived exosomes could resist toxin‐induced liver damage by activating proliferative and regenerative responses.^74^ In addition, exosomes released by chorionic plate‐derived mesenchymal stem cells (CP‐MSCs) and adipose‐derived mesenchymal stem cells (AMSCs) can be potentially used in the treatment of liver injury.^75^, ^76^

### 4.5 Viral hepatitis

#### 4.5.1 Biomarkers

Studies have shown that exosomes are associated with the spread of hepatitis virus. Exosomes secreted by infected cells contain viral nucleic acids and proteins. Viruses can spread to surrounding cells and cause infections through hepatocyte‐derived exosomes.^77^, ^78^, ^79^ Li et al showed that, in patients with CHB infection, who have normal alanine aminotransferase (ALT) levels, 18 types of exosomal miRNAs were up‐regulated, including hsa‐miR‐221‐3p and hsa‐miR‐25‐3p. In addition, six types of exosomal miRNAs were down‐regulated, including hsa‐miR‐372‐3p and hsa‐miR‐10a‐5p, and these exosomal miRNAs were more sensitive indicators of liver inflammation than ALT.^80^ In certain studies on HCV infection, researchers have compared the miRNA expression in serum exosomes between patients with chronic hepatitis C (CHC) and normal controls, and found that the miRNA expression patterns are related to liver fibrosis stage and inflammation grade in patients with CHC infection. Exosomal miRNAs may serve as biomarkers for staging liver disease.^81^ Bukong et al isolated exosomes from the serum samples of patients with CHC, which contain replicating components of viral RNA associated with miR‐122, Argonaute 2, and HSP90. These exosomes can infect human primary hepatocytes as well as human liver cancer Huh7 cells and can therefore be used as potential biomarkers.^82^

#### 4.5.2 Treatment

**Figure 4:**
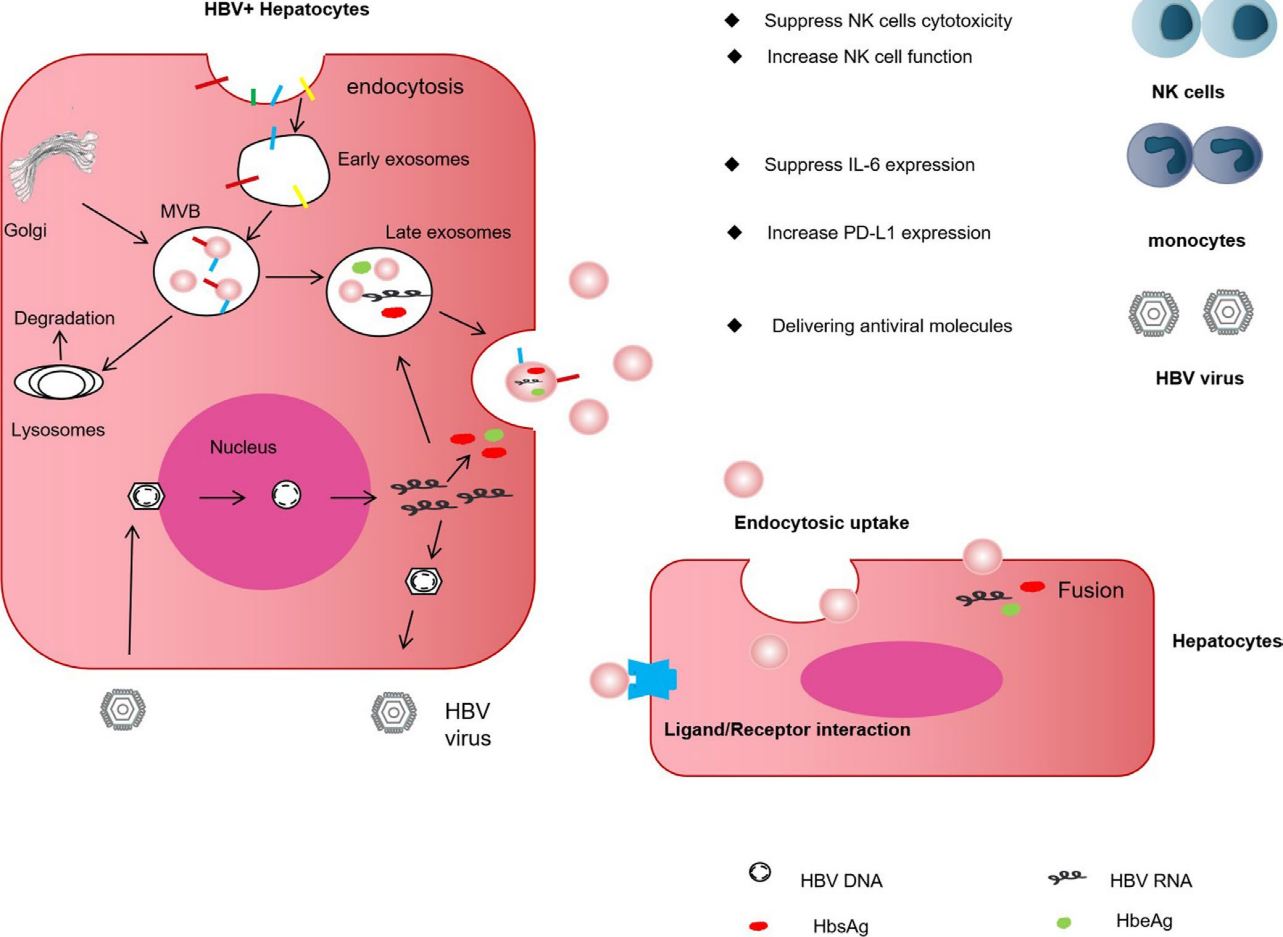
Exosomes and hepatitis B virus (HBV) infection. During chronic HBV infection, infected hepatocytes can release exosomes that can exhibit contrasting actions. Exosomes contain HBV proteins and nucleic acids, which can promote as well as suppress HBV infection. IFN‐γ, interferon‐γ; TNF‐α, tumour necrosis factor‐α; RIG‐1, retinoic acid‐inducible gene I; NF‐κB, nuclear factor κB

During hepatitis B virus (HBV) infection, exosomes affect cytokine‐mediated signalling pathways by suppressing immune responses and promoting infection. In contrast, they also play an anti‐infective role by enhancing the functions of macrophages and NK cells and transmitting antiviral molecules^83^ (Figure 4). For example, IFN‐α stimulates the release of exosomes from non‐parenchymal liver cells (resistant to viral infection), which can be internalized by hepatocytes, which subsequently exhibit antiviral properties against HBV.^84^, ^85^ This provides the theoretical basis for exosome‐mediated immunotherapy of HBV infection. In addition, exosomes activate immune responses and serve as an attractive adjuvant. For example, exosomes isolated from LPS/endotoxin‐stimulated human monocytes were observed to promote inflammatory responses in healthy mice by inducing cytokine production. When these exosomes were used as adjuvants of hepatitis B surface antigen (HBsAg), they were observed to induce a cellular immune response in mice.^86^ Genetically engineered exosomes play a more significant role in resisting HBV infection. Researchers have constructed a DNA vector expressing the mutant form of the exosome anchoring protein Nef and specific antigens and injected it into animals to produce engineered exosomes, which induced specific cytotoxic T lymphocyte‐mediated immunity against HBV. Activated HBV‐specific cytotoxic T lymphocytes were observed to exert important therapeutic effects in HBV infection; such genetically engineered exosomes may serve as novel HBV vaccines.^87^

Exosomes are also used for the treatment of HCV infection. Deng et al showed that syntenin is a protein involved in the secretory pathway of exosomes. In vitro, syntenin overexpression was observed to induce the release of exosomes containing the HCV protein E2. These exosomes may act as antibody baits during HCV infection.^77^

## 5 SUMMARY

Exosomes are important tools in cell‐to‐cell communication. Compared with current diagnostic agents, exosomes can be used a non‐invasive agents that do not cause major side effects. Despite their limitations, exosomes are considered promising screening and treatment tools in liver diseases. This review aimed to describe the functions of exosomes derived from different liver cells and their role in different liver diseases. At present, with the increase in research on exosomes, the gradual increase in their demand as biological carriers has been observed. Further research on the role of exosomes in liver diseases will improve the diagnosis and treatment of liver diseases.

## CONFLICT OF INTEREST

The authors report no conflicts of interest.

## AUTHOR CONTRIBUTIONS

**Yan Jiao:** Writing‐original draft (equal). **Xu Ping:** Software (equal). **Shi Honglin:** Project administration (equal). **Dexi Chen:** Writing‐review & editing (equal). **Hongbo Shi:** Writing‐review & editing (equal).

## Data Availability

Data sharing not applicable—no new data generated, or the article describes entirely theoretical research.
